# Supplementary material for: Partial freezing of rat livers extends preservation time by 5-fold
Source: Nat Commun. 2022 Jul 15;13:4008. doi: 10.1038/s41467-022-31490-2 (PMC9287450; doi:10.1038/s41467-022-31490-2)
Supplement: Supplementary file 1 — Supplementary Information [file 41467_2022_31490_MOESM1_ESM.pdf]

SUPPLEMENTAL FIGURES AND TABLES

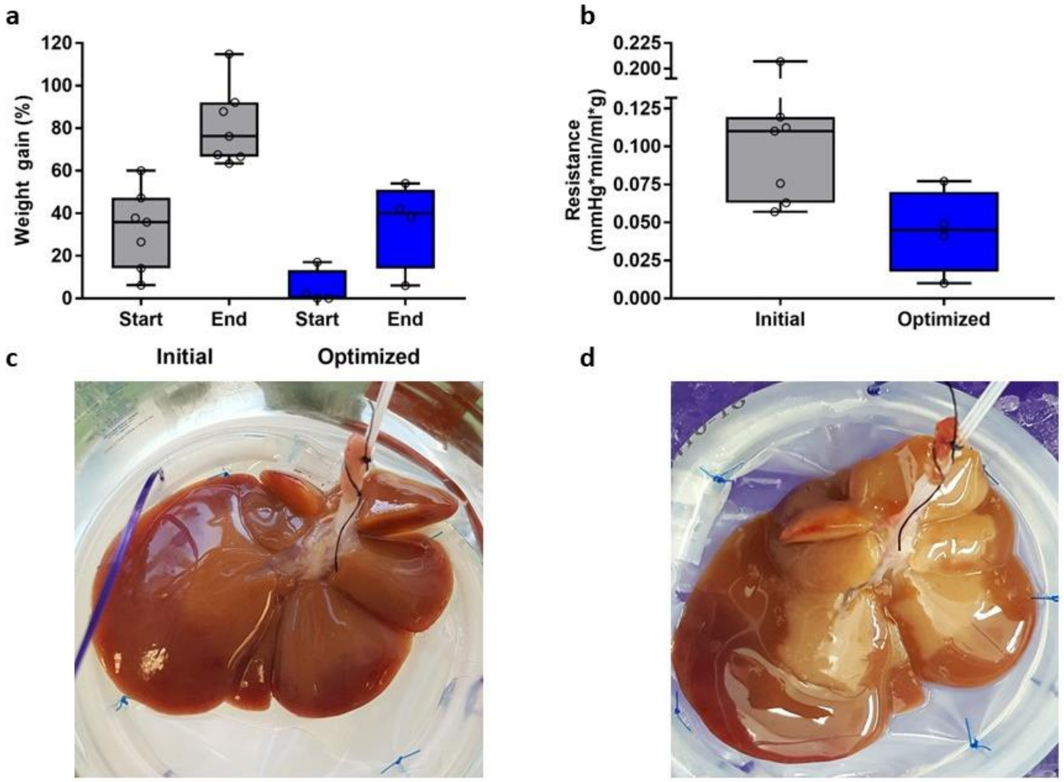

Supplementary Figure S1: Optimization of the (un)loading of cryoprotective agents improves gross outcomes after partial freezing. **a)** Weight gain during SNMP recovery during the initial partial freezing protocol (grey) and the optimized protocol (blue). **b)** Vascular resistance between the portal vein and inferior vena cava during SNMP recovery during the initial partial freezing protocol (grey) and the optimized protocol (blue). **c)** Photo showing inhomogeneous preconditioning during with the initial protocol. **d)** Photo showing the extensive and inhomogeneous injury after partial freezing using the initial protocol. Source data are provided as a Source Data file.

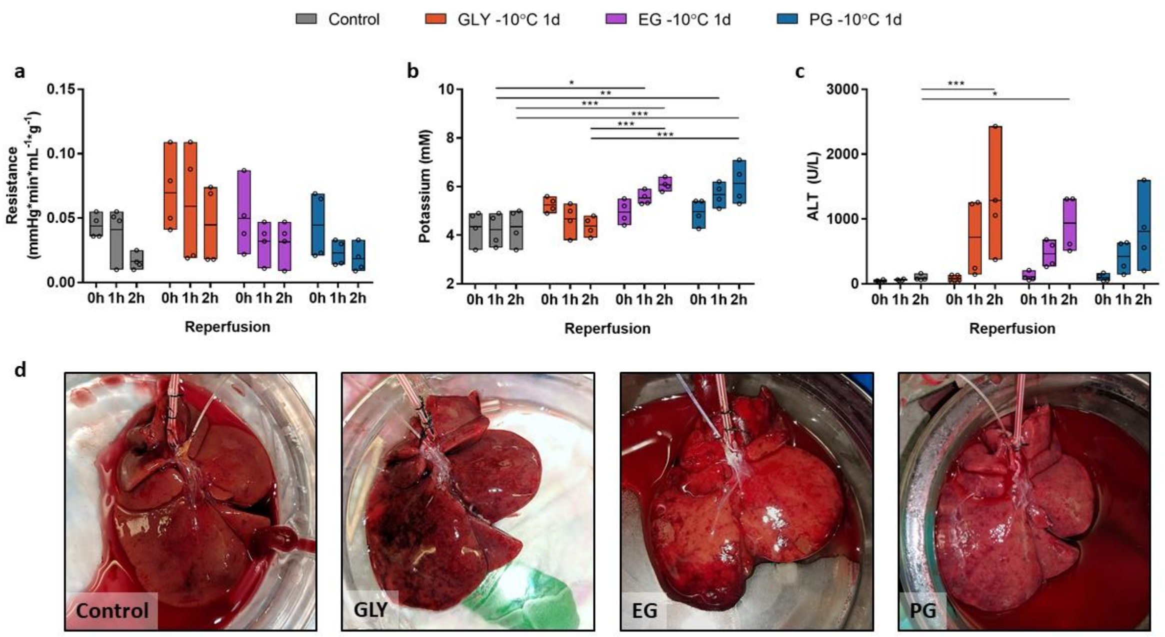

Supplementary Figure S2: Effect of permeating cryoprotective agents on liver function and injury after partial freezing at -10°C for 1 day. **a)** Vascular resistance between the portal vein and the intrahepatic vena cava. **b)** Potassium concentration in the intrahepatic vena cava (IVC). **c)** Alanine aminotransferase concentration (ALT) in the IVC. **d)** Photos of the livers at the end of simulated transplantation. Controls (grey)= 1 day hypothermic preservation, GLY= glycerol (red), EG = ethylene glycol (purple), PG = propylene glycol (dark blue). Stars denote statistical significance (two-way ANOVA, followed by Tukey's post-hoc test): *0.01 < p < 0.05; **0.001 < p < 0.01; ***0.0001 < p < 0.001; ****p < 0.0001. Boxes: Floating bars (min to max), with a line at the mean. Source data are provided as a Source Data file.

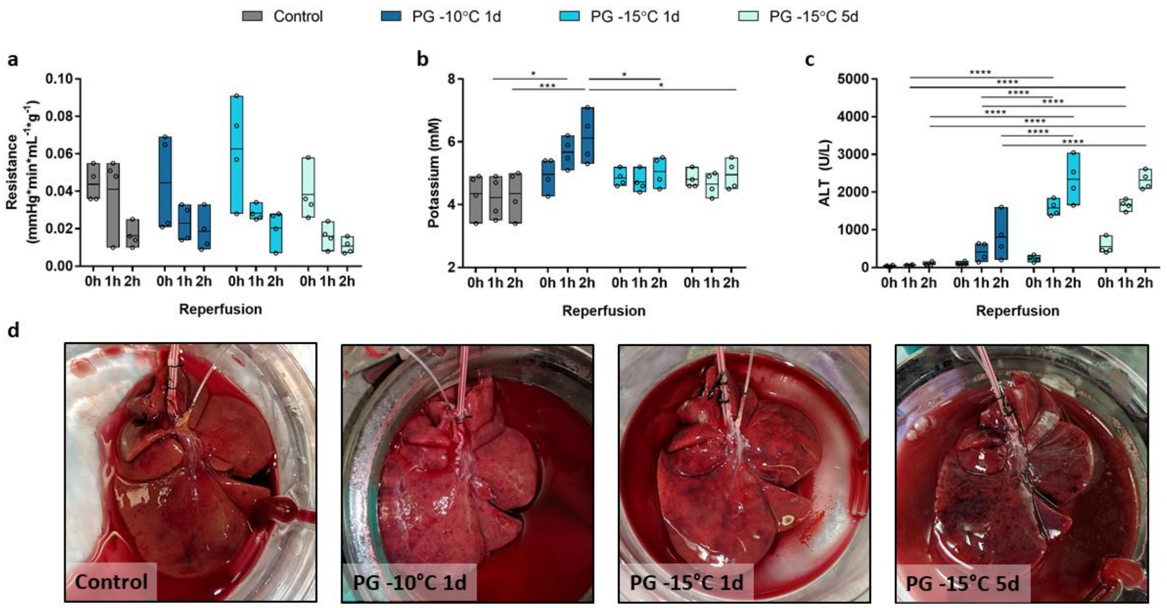

Supplementary Figure S3: Effect of storage temperature (-10 vs. -15°C) and duration of storage (1 vs. 5 days) on liver function and injury after partial freezing with propylene glycol. **a)** Vascular resistance between the portal vein and the intrahepatic vena cava. **b)** Potassium concentration in the intrahepatic vena cava (IVC). **c)** Alanine aminotransferase concentration (ALT) in the IVC. **d)** Photos of the livers at the end of simulated transplantation. Controls (grey) = 1 day hypothermic preservation, PG = propylene glycol stored for 1 day at -10°C (dark blue), 1 day at -15°C (blue), and 5 days at -15°C (light blue). Stars denote statistical significance (two-way ANOVA, followed by Tukey's post-hoc test): *0.01 < p < 0.05; **0.001 < p < 0.01; ***0.0001 < p < 0.001; ****p < 0.0001. Boxes: Floating bars (min to max), with a line at the mean. Source data are provided as a Source Data file.

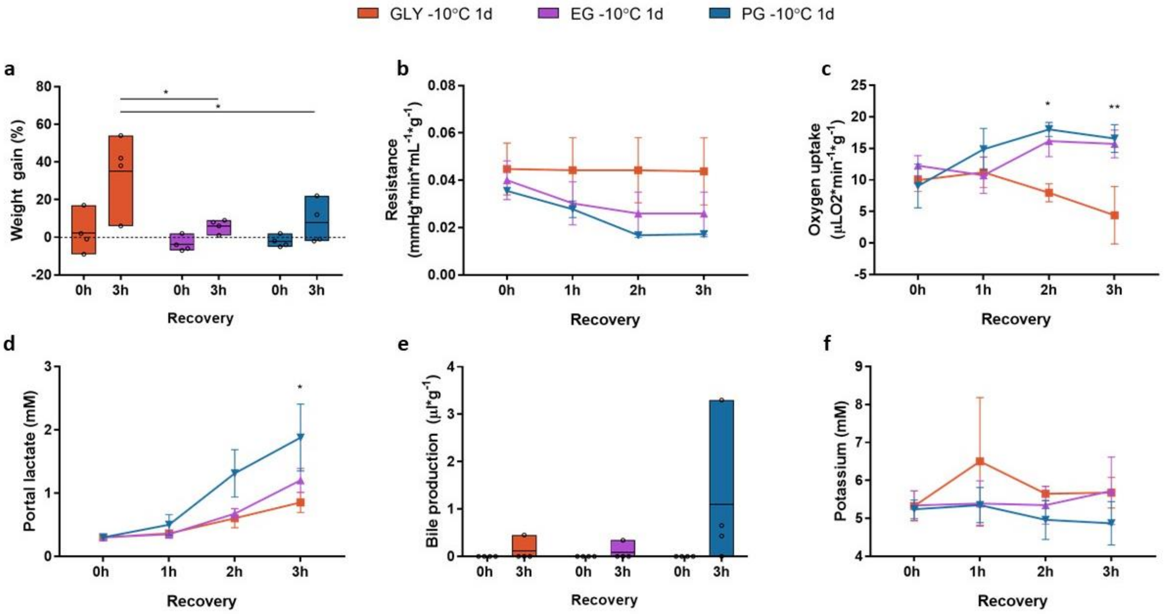

Supplementary Figure S4: Effect of permeating cryoprotective agents on liver viability and injury during subnormothermic recovery. **a)** Weight gain as percentage of the procurement weight. **b)** Vascular resistance between the portal vein and the intrahepatic vena cava. **c)** Oxygen uptake. **d)** Lactate concentration in the portal vein. **e)** Bile production. **f)** Potassium concentration in the intrahepatic vena cava. GLY= glycerol (red), EG = ethylene glycol (purple), PG = propylene glycol (dark blue). Stars denote statistical significance (two-way ANOVA, followed by Tukey's post-hoc test): *0.01 < p < 0.05; **0.001 < p < 0.01; ***0.0001 < p < 0.001; ****p < 0.0001. Boxes: Floating bars (min to max), with a line at the mean. Lines: mean, error bars: SEM. Source data are provided as a Source Data file.

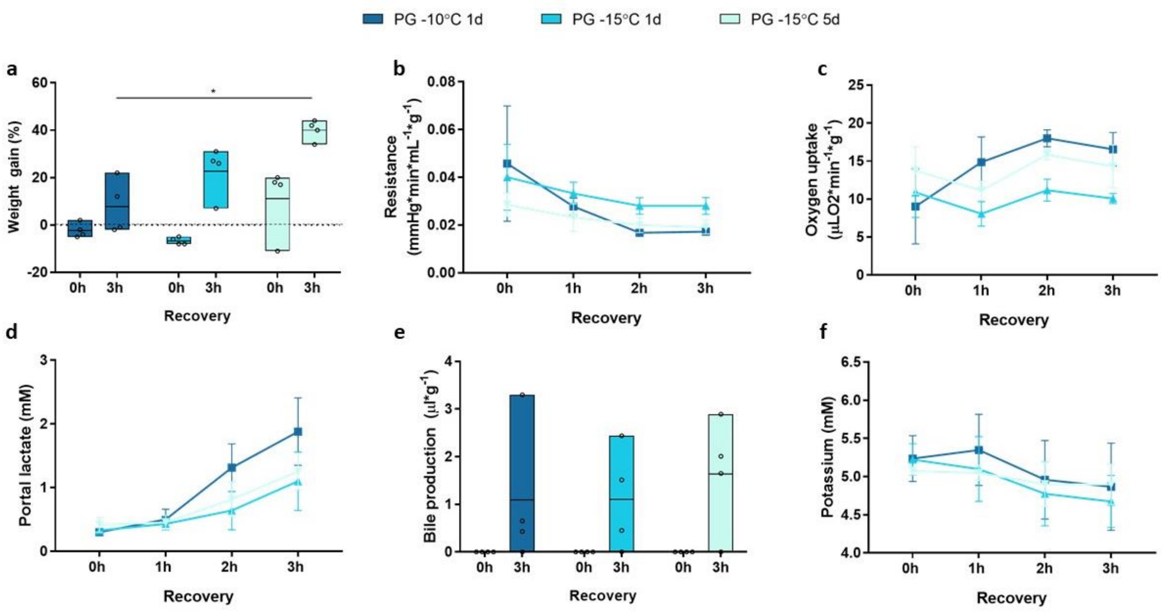

Supplementary Figure S5: Effect of freezing temperature and storage duration on liver viability and injury during subnormothermic recovery. **a)** Weight gain as percentage of the procurement weight. **b)** Vascular resistance between the portal vein and the intrahepatic vena cava. **c)** Oxygen uptake. **d)** Lactate concentration in the portal vein. **e)** Bile production. **f)** Potassium concentration in the intrahepatic vena cava. PG = propylene glycol stored for 1 day at -10°C (dark blue), 1 day at -15°C (blue), and 5 days at -15°C (light blue). Stars denote statistical significance (two-way ANOVA, followed by Tukey's post-hoc test): *0.01 < p < 0.05; **0.001 < p < 0.01; ***0.0001 < p < 0.001; ****p < 0.0001. Boxes: Floating bars (min to max), with a line at the mean. Lines: mean, error bars: SEM. Source data are provided as a Source Data file.

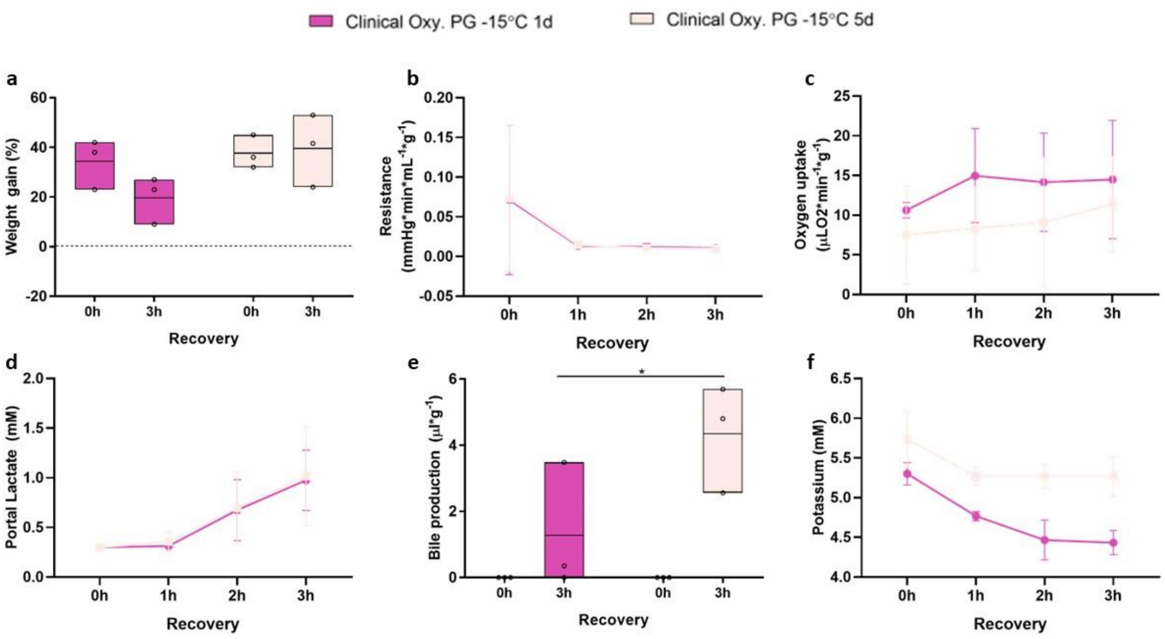

Supplementary Figure S6: Effect of a clinical grade oxygenator on liver viability and injury during subnormothermic recovery. **a)** Weight gain as percentage of the procurement weight. **b)** Vascular resistance between the portal vein and the intrahepatic vena cava. **c)** Oxygen uptake. **d)** Lactate concentration in the portal vein. **e)** Bile production. **f)** Potassium concentration in the intrahepatic vena cava. PG = propylene glycol stored for 1 day at -15°C (dark pink) or 5 days at -15°C (light pink) perfused using a clinical grade oxygenator. Stars denote statistical significance (two-way ANOVA, followed by Tukey's post-hoc test): *$0.01 < p < 0.05$; **$0.001 < p < 0.01$; ***$0.0001 < p < 0.001$; ****$p < 0.0001$. Boxes: Floating bars (min to max), with a line at the mean. Lines: mean, error bars: SEM. Source data are provided as a Source Data file.

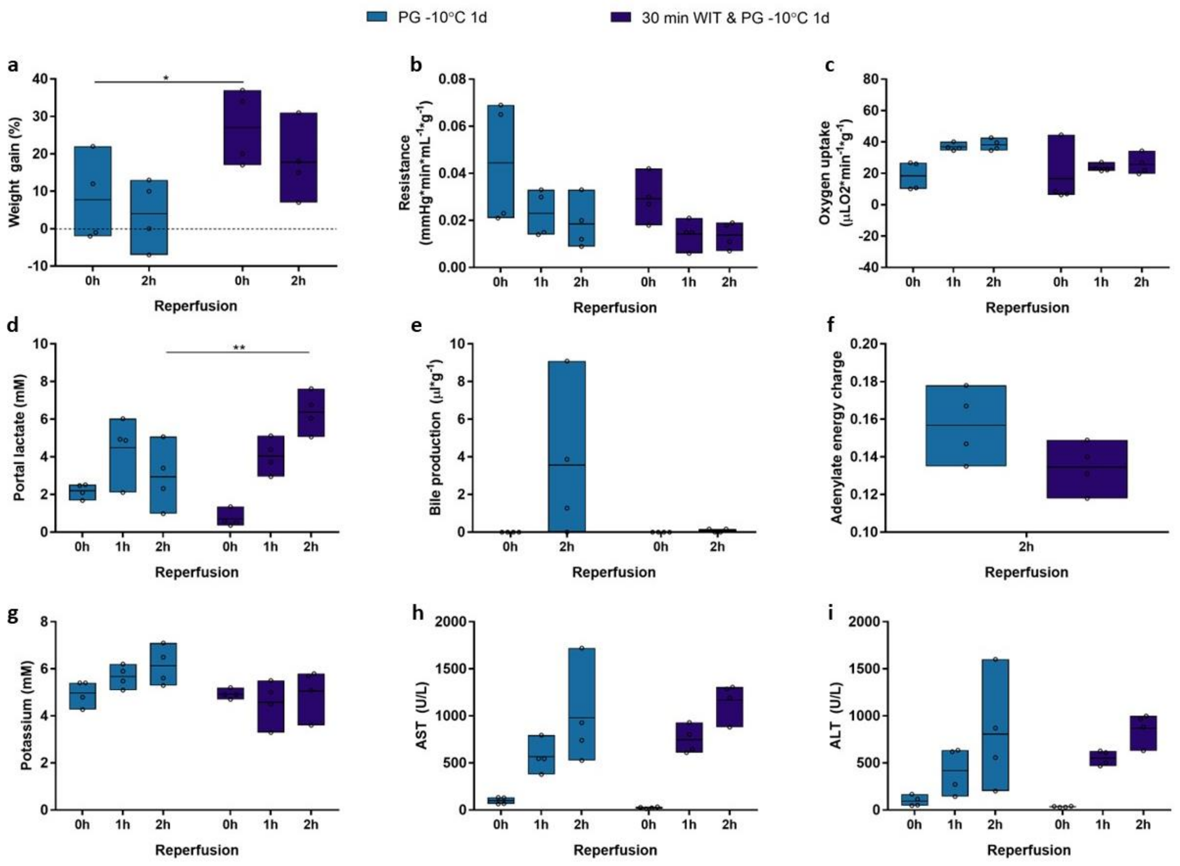

Supplementary Figure S7: Effect of 30 minutes warm ischemia time on liver viability and injury after partial freezing with propylene glycol at -10°C for 1 day. **a)** Weight gain as percentage of the procurement weight. **b)** Vascular resistance between the portal vein and the intrahepatic vena cava. **c)** Oxygen uptake. **d)** Lactate concentration in the portal vein. **e)** Bile production. **f)** Tissue adenylate energy charge. **g)** Potassium concentration in the intrahepatic vena cava. **h)** Aspartate aminotransferase concentration (AST) in the IVC. **i)** Alanine aminotransferase concentration (ALT) in the IVC. PG = propylene glycol (dark blue), 30 min WIT = 30 minutes warm ischemia time (dark purple). Stars denote statistical significance (two-way ANOVA, followed by Tukey's post-hoc test): *0.01 < p < 0.05; **0.001 < p < 0.01; ***0.0001 < p < 0.001; ****p < 0.0001. Boxes: Floating bars (min to max), with a line at the mean. Source data are provided as a Source Data file.

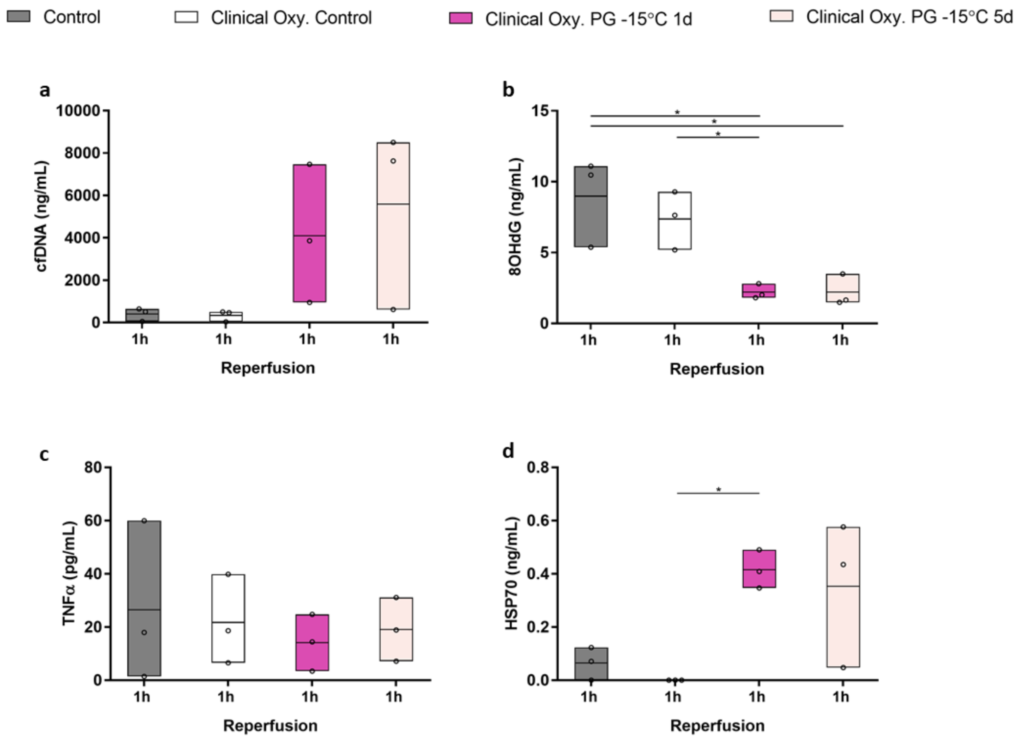

Supplementary Figure S8: Effect of a clinical grade oxygenator on damage-associated molecular patterns (DAMPs) after partial freezing with propylene glycol at -15°C for 1 and 5 days. **a)** cell-free DNA (cfDNA). **b)** 8-oxo-2'-deoxyguanosine (8-OHdG). **c)** Tumor Necrosis Factor α (TNFα). **d)** Heat Shock Protein 70 (HSP70). Control (grey) = 1 day hypothermic preservation, Clinical Oxy. Control (white) = 1 day hypothermic preservation perfused using a clinical grade oxygenator, PG = propylene glycol stored for 1 day at -15°C (dark pink) or 5 days at -15°C (light pink) perfused using a clinical grade oxygenator. Stars denote statistical significance (one-way ANOVA, followed by Tukey's post-hoc test): *0.01 < p < 0.05; **0.001 < p < 0.01; ***0.0001 < p < 0.001; ****p < 0.0001. Boxes: Floating bars (min to max), with a line at the mean. Source data are provided as a Source Data file.

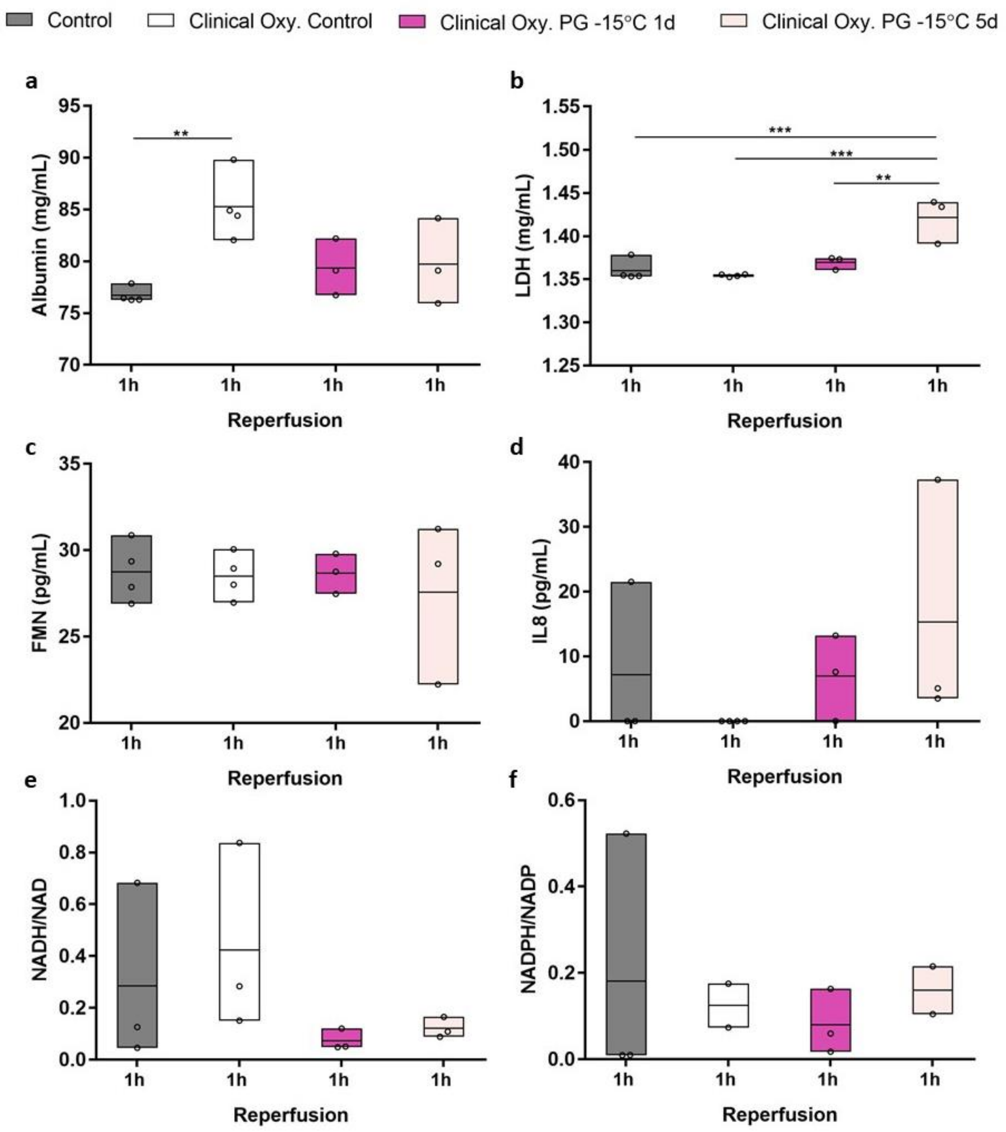

Supplementary Figure S9: Effect of a clinical grade oxygenator on liver viability markers after partial freezing with propylene glycol at -15°C for 1 and 5 days. **a)** Albumin. **b)** Lactate Dehydrogenase (LDH). **c)** Flavin Mononucleotide (FMN). **d)** Interleukin-8 (IL8). **e)** Nicotinamide adenine dinucleotide (NADH/NAD) ratio. **f)** Nicotinamide adenine dinucleotide phosphate (NADPH/NADP) ratio. Albumin, LDH, FMN, and IL-8 were measured from the perfusate, while NAD, NADH, NADP, NADPH were measured from tissue. Control (grey) = 1 day hypothermic preservation, Clinical Oxy. Control (white) = 1 day hypothermic preservation perfused using a clinical grade oxygenator, PG = propylene glycol stored for 1 day at -15°C (dark pink) or 5 days at -15°C (light pink) perfused using a clinical grade oxygenator. Stars denote statistical significance (one-way ANOVA, followed by Tukey's post-hoc test): *$0.01 < p < 0.05$; **$0.001 < p < 0.01$; ***$0.0001 < p < 0.001$; ****$p < 0.0001$. Boxes: Floating bars (min to max), with a line at the mean. Source data are provided as a Source Data file.

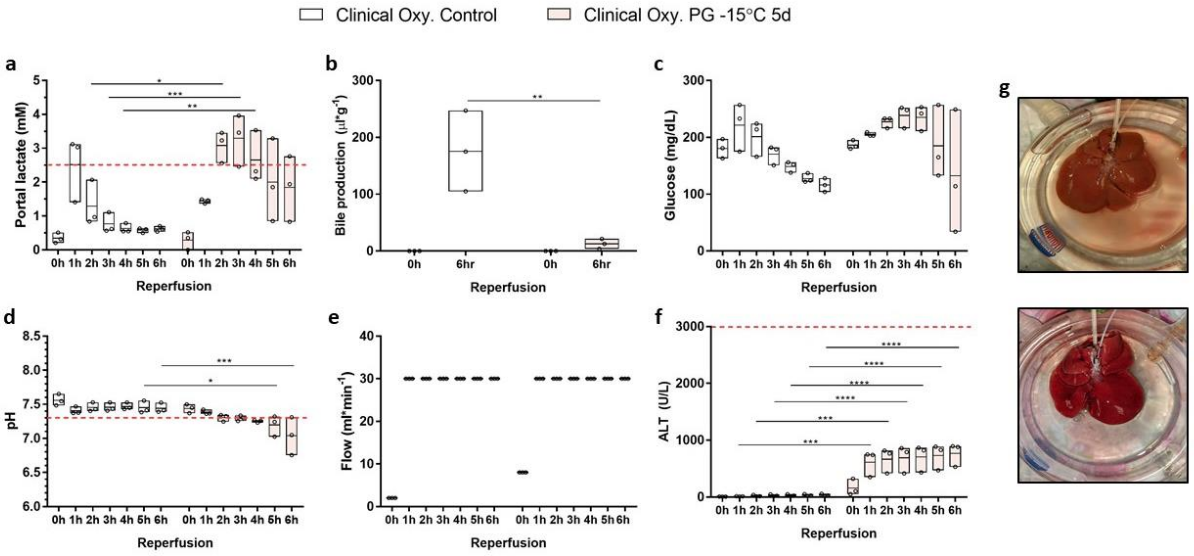

Supplementary Figure S10: Effect of 6-hour simulated transplantation in the presence of whole blood with a clinical grade oxygenator on liver viability markers after partial freezing with propylene glycol at -15°C for 5 days. **a)** Lactate concentration in the portal vein. **b)** Bile production. **c)** Glucose concentration in the portal vein. **d)** pH in the portal vein. e**)** Portal flow rate. f**)** Alanine aminotransferase concentration (ALT) in the IVC. **g)** Photos of the livers at the end of simulated transplantation. Clinical Oxy. Control (white) = 1-day hypothermic preservation perfused using a clinical grade oxygenator, PG = propylene glycol stored for 5 days at -15°C (light pink) perfused using a clinical grade oxygenator. Stars denote statistical significance (two-way ANOVA, followed by Tukey's post-hoc test): *0.01 < p < 0.05; **0.001 < p < 0.01; ***0.0001 < p < 0.001; ****p < 0.0001. Boxes: Floating bars (min to max), with a line at the mean. Where applicable, red dotted lines indicate the clinical benchmark. Source data are provided as a Source Data file.

Supplementary Table S1: Composition of all solutions used in each phase of the partial freezing protocol. *present in UW. BSA = bovine serum albumin, PEG = polyethylene glycol, HES = hydroxyethyl starch, GLY = glycerol, EG = ethylene glycol, PG = propylene glycol.

	**Subnormothermic Preconditioning solution**	**Hypothermic Preloading solution**	**Storage Solution**	**Thawing Solution**	**Recovery Solution**	**Whole Blood Solution**
Base solution	**WE**	**WE**	**UW**	**WE**	**WE**	**WE**
Total volume	**250 ml**	**250 ml**	**100 ml**	**250 ml**	**500 ml**	**100 ml**
Additives						
Insulin	200 U/l	200 U/l	10 U/l	20 U/l	20 U/l	20 U/l
Heparin	10,000 U/l	4,000 U/l	-	4,000 U/l	5,000 U/l	5,000 U/l
Dexamethasone	24 mg/l	24 mg/l	24 mg/l	24 mg/l	24 mg/l	24 mg/l
Hydrocortisone	25 mg/ml	25 mg/ml	-	25 mg/ml	25 mg/ml	25 mg/ml
Penicillin	40,000 ug/l	40,000 ug/l	-	40,000 ug/l	40,000 ug/l	40,000 ug/l
Streptomycin	40,000 U/l	40,000 U/l	-	40,000 U/l	40,000 U/l	40,000 U/l
Glutathione	-	-	*0.922 g/l**	1.536 g/l	1.536 g/l	-
Blood						
Fresh whole rat blood	-	-	-	-	-	~10-12 ml/l
Macromolecules						
BSA	10 g/l	10 g/l	-	10 g/l	10 g/l	10 g/l
35 kDa PEG	20 g/l	20 g/l	50 g/l	20 g/l	20 g/l	-
HES	-	30 g/l	*50 g/l**	30 g/l	-	-
Saccharides						
3-O-Methyl D glucose	19.42 g/l	19.42 g/l	19.42 g/l	19.42 g/l	-	-
Raffinose	-	15.12 g/l	*17,83 g/l**	15.12 g/l	-	-
Trehalose	-	-	18.92 g/l	18.92 g/l	-	-
Permeating CPA						
GLY, EG, or PG	-	60 ml/l	120 ml/l	60 ml/l	-	-
Ice nucleating agent						
Snomax	-	-	1 g/l	-	-	-

Supplementary Table S2: List of suppliers for each reagent used in partial freezing solutions.

Reagent	*Supplier*
University of Wisconsin (UW) solution	Bridge to Life
Williams' Medium E	Sigma-Aldrich
Insulin (Humulin R)	MGH pharmacy
Sodium heparin	MGH pharmacy
Dexamethasone	Sigma-Aldrich
Hydrocortisone	MGH pharmacy
Penicillin-Streptomycin	Invitrogen
L-Glutathione	Sigma-Aldrich
Bovine Serum Albumin (BSA)	Sigma-Aldrich
35kDa Polyethylene glycol (PEG)	Sigma-Aldrich
Hydroxyethyl starch 130/0.4 (HES)	AK Scientific
3–O–methyl glucose	Chem-Impex
D-Raffinose pentahydrate	Chem-Impex
D-(+)-Trehalose dihydrate	Sigma-Aldrich
Glycerol (CAS No. 56-81-5)	Fisher Scientific
Ethylene Glycol (CAS No. 107-21-1)	Sigma-Aldrich
Propylene Glycol (CAS No. 57-55-6)	Fisher Scientific
Snomax	Telemet
