# Supplementary material for: Partial freezing of rat livers extends preservation time by 5-fold
Source: Nat Commun. 2022 Jul 15;13:4008. doi: 10.1038/s41467-022-31490-2 (PMC9287450; doi:10.1038/s41467-022-31490-2)
Supplement: Supplementary file 2 — Reporting Summary [file 41467_2022_31490_MOESM2_ESM.pdf]

nature research

Corresponding author(s): Mehmet Toner

Last updated by author(s): May 31, 2022

Reporting Summary

Nature Research wishes to improve the reproducibility of the work that we publish. This form provides structure for consistency and transparency in reporting. For further information on Nature Research policies, see our Editorial Policies and the Editorial Policy Checklist.

Statistics

For all statistical analyses, confirm that the following items are present in the figure legend, table legend, main text, or Methods section.

n/a	Confirmed	
☐	☒	The exact sample size (n) for each experimental group/condition, given as a discrete number and unit of measurement
☐	☒	A statement on whether measurements were taken from distinct samples or whether the same sample was measured repeatedly
☐	☒	The statistical test(s) used AND whether they are one- or two-sided<br>*Only common tests should be described solely by name; describe more complex techniques in the Methods section.*
☒	☐	A description of all covariates tested
☐	☒	A description of any assumptions or corrections, such as tests of normality and adjustment for multiple comparisons
☐	☒	A full description of the statistical parameters including central tendency (e.g. means) or other basic estimates (e.g. regression coefficient) AND variation (e.g. standard deviation) or associated estimates of uncertainty (e.g. confidence intervals)
☐	☒	For null hypothesis testing, the test statistic (e.g. F, t, r) with confidence intervals, effect sizes, degrees of freedom and P value noted<br>*Give P values as exact values whenever suitable.*
☒	☐	For Bayesian analysis, information on the choice of priors and Markov chain Monte Carlo settings
☒	☐	For hierarchical and complex designs, identification of the appropriate level for tests and full reporting of outcomes
☒	☐	Estimates of effect sizes (e.g. Cohen's d, Pearson's r), indicating how they were calculated

Our web collection on statistics for biologists contains articles on many of the points above.

Software and code

Policy information about availability of computer code

Data collection	Data was collected in Microsoft Excel 365, Aperio ImageScope (Leica Biosystems)
Data analysis	Data analysis was performed in GraphPad Prism 7.03

For manuscripts utilizing custom algorithms or software that are central to the research but not yet described in published literature, software must be made available to editors and reviewers. We strongly encourage code deposition in a community repository (e.g. GitHub). See the Nature Research guidelines for submitting code & software for further information.

Data

Policy information about availability of data

All manuscripts must include a data availability statement. This statement should provide the following information, where applicable:

- Accession codes, unique identifiers, or web links for publicly available datasets
- A list of figures that have associated raw data
- A description of any restrictions on data availability

The authors declare that the data supporting the findings of this study are available within the paper and its supplementary information files. Further, source data are provided with this paper. Any additional data, if needed, will be provided upon request.

Field-specific reporting

Life sciences study design

All studies must disclose on these points even when the disclosure is negative.

Sample size	The goal of this proof of concept study was to demonstrate that subzero organ preservation by partial freezing of rat livers is possible. We used 4 livers in each experimental and control group to demonstrate feasibility and repeatability of partial freezing. This sample size was selected based on prior data whereby n=4 provides 80% power at one-sided alpha of 0.05.
Data exclusions	No data were exluded
Replication	Each experirment was repeated 4 times to verify reproducability. Attempts at replication that suffered from surgical failure during procurement or equipment failure during perfusion or freezing were exluded. All other attempts at replication were succesfull and included in the article.
Randomization	All livers and samples were randomly allocated to the experimental groups. For logistical reasons true randomization was not possible.
Blinding	Blinding was performed for all histological analysis. For logistical reasons blinding was not always possible.

Reporting for specific materials, systems and methods

We require information from authors about some types of materials, experimental systems and methods used in many studies. Here, indicate whether each material, system or method listed is relevant to your study. If you are not sure if a list item applies to your research, read the appropriate section before selecting a response.

Materials & experimental systems

n/a	Involved in the study	
☐	☒	Antibodies
☒	☐	Eukaryotic cell lines
☒	☐	Palaeontology and archaeology
☐	☒	Animals and other organisms
☒	☐	Human research participants
☒	☐	Clinical data
☒	☐	Dual use research of concern

Methods

n/a	Involved in the study	
☒	☐	ChIP-seq
☒	☐	Flow cytometry
☒	☐	MRI-based neuroimaging

Antibodies

Antibodies used	GLUT-2 Proteintech cat # 20436-1-AP
Validation	This is a antibody with reactivity to human, with verified application for IHC from the manufacturer. Negative controls were used to ensure antibody specificity.

Animals and other organisms

Policy information about studies involving animals; ARRIVE guidelines recommended for reporting animal research

Laboratory animals	The experimental protocol was approved by the Institutional Animal Care and Use Committee (IACUC) of Massachusetts General Hospital (Boston, MA, USA; 2017N000227). Lewis rats were socially housed in temperature (70F +/- 2F) and humidity (30-70%) controlled environments within pathogen free HEPA filtered ventilated cages, with alternating 12 hour light/dark cycles. Animals were provided sterilized standard rat chow and water ad libitum. Livers were procured from male rats with a body weight of 250-300 g and age age 10-12 weeks. Laboratory animals were supplied by Charles River Laboratories, Wilmington, MA, USA.
Wild animals	This study did not involve wild animals
Field-collected samples	This study did not involve samples collected from the field
Ethics oversight	The health and welfare of the animals was maintained by the Massachusetts General Hospital Center of Comparative Medicine (CCM). The experimental protocols were approved by the Institutional Animal Care and Use Committee (IACUC) of Massachusetts General Hospital (Boston, MA, USA).

Note that full information on the approval of the study protocol must also be provided in the manuscript.
